# Droplets for Gene Editing Using CRISPR-Cas9 and Clonal Selection Improvement Using Hydrogels

**DOI:** 10.3390/mi15030413

**Published:** 2024-03-19

**Authors:** Camilo Pérez-Sosa, Maximiliano S. Pérez, Alexander Paolo Vallejo-Janeta, Shekhar Bhansali, Santiago Miriuka, Betiana Lerner

**Affiliations:** 1 IREN Center, National Technological University, Buenos Aires B1706EAH, Argentina; camilo.bm91@gmail.com (C.P.-S.); pvallejoj@gmail.com (A.P.V.-J.); 2 Institute of Biomedical Engineering, Buenos Aires University (UBA), Buenos Aires C1063ACV, Argentina; 3 Collaborative Research Institute Intelligent Oncology (CRIION), Hermann-Herder-Straße 4, 79104 Freiburg im Breisgau, Germany; 4 Department of Electrical and Computer Engineering, Florida International University, Miami, FL 33174, USA; sbhansali@gmail.com; 5 National Council for Scientific and Technical Research—Foundation for the Fight against Neurological Diseases of Childhood, (LIAN-CONICET-FLENI), FLENI Sede Escobar, Ruta 9 Km 53, Belén de Escobar, Buenos Aires B1625XAF, Argentina; smiriuka@fleni.org.ar

**Keywords:** microfluidics, single cell, droplets, CRISPR-Cas9, clone selection

## Abstract

Gene editing tools have triggered a revolutionary transformation in the realms of cellular and molecular physiology, serving as a fundamental cornerstone for the evolution of disease models and assays in cell culture reactions, marked by various enhancements. Concurrently, microfluidics has emerged over recent decades as a versatile technology capable of elevating performance and reducing costs in daily experiments across diverse scientific disciplines, with a pronounced impact on cell biology. The amalgamation of these groundbreaking techniques holds the potential to amplify the generation of stable cell lines and the production of extracellular matrix hydrogels. These hydrogels, assuming a pivotal role in isolating cells at the single-cell level, facilitate a myriad of analyses. This study presents a novel method that seamlessly integrates CRISPR-Cas9 gene editing techniques with single-cell isolation methods in induced pluripotent stem cell (hiPSC) lines, utilizing the combined power of droplets and hydrogels. This innovative approach is designed to optimize clonal selection, thereby concurrently reducing costs and the time required for generating a stable genetically modified cell line. By bridging the advancements in gene editing and microfluidic technologies, our approach not only holds significant promise for the development of disease models and assays but also addresses the crucial need for efficient single-cell isolation. This integration contributes to streamlining processes, making it a transformative method with implications for enhancing the efficiency and cost-effectiveness of stable cell line generation. As we navigate the intersection of gene editing and microfluidics, our study marks a significant stride toward innovative methodologies in the dynamic landscape of cellular and molecular physiology research.

## 1. Introduction

Editing mechanisms for genes assume a crucial role across different domains, encompassing fundamental science, applied research, and personalized medicine [1,2,3,4]. The broad applications of these tools prompted the development of numerous strategies employing diverse methodologies to enhance performance and reduce costs associated with genome modifications in cell lines.

CRISPR technology, on the other hand, has garnered immense popularity owing to its specificity in introducing point mutations within the genome and its cost advantages over comparable methods like zinc-finger technology or viral-based modifications. This revolutionary tool holds immense potential for various fields, including medicine, agriculture, and biotechnology. While the functions of CRISPR have been understood for decades in bacteria, it was not until 2013 that the Cas-9 enzyme was adapted for routine gene editing in eukaryotic cells [5,6].

For gene editing using CRISPR, two key components are considered: CRISPR guide RNA (gRNA) and Cas protein. CRISPR gRNA leads the enzyme to the specific location in the genome that needs editing, and Cas protein is the enzyme that acts as the molecular scissors cutting the DNA signaled by the gRNA [7]. There is a whole family of proteins within the CRISPR-Cas system [8]; in bacteria, Cas proteins exhibit distinct functionalities; some play a role in their immune system, and others work as RNA-binding proteins [9]. In the context of gene editing, the Cas technology mainly refers to the use of Cas9.

Cas9 protein is an RNA-guided nuclease that, once bound to the target site, introduces a precise double-stranded cut in the DNA. This targeted cleavage initiates the cellular repair mechanisms, paving the way for the introduction of desired genetic modifications (e.g., activation or knock-out of genes) [10,11]. As this enzyme is RNA-guided, the specificity of this method significantly reduces the risk of unintended edits compared to previous gene editing methods [12].

The potential of CRISPR technology is vast, as recent studies showcased its precision in generating point mutations, positioning it as one of the most revolutionary techniques in applied biomedical sciences [13]. Within the advantages of CRISPR technology, we can mention the specificity in targeting DNA sequences, the efficiency in the addition of multiple genes simultaneously, and the affordability of implementation [14]. Nonetheless, challenges persist, particularly in the efficiency and efficacy of mutating apparently homogeneous stable cell lines. Recent discoveries reveal substantial differences in gene expression among cells of the same tissue or cell line, modifying their behavior and limiting efficient genome editing [15].

This challenge becomes more evident in human induced pluripotent stem cell (hiPSC) lines, where genomic heterogeneity was reported due to independently reprogrammed expression in each cell [16]. This poses difficulties in selecting clones for stable lines through antibiotic and random selection methods, thereby increasing experimental time.

In response to these challenges, fluid technology, particularly microfluidic technology [17,18], addressed various limitations and increased throughput. Droplet technology gained prominence in cell biology, enabling the isolation, classification, and specific editing of individual cells using different techniques. This facilitates the use of various plasmids in both transient and non-transient manners, followed by selection for diverse applications, such as single-cell culture using biocompatible hydrogels or extracellular matrix compounds [19,20,21,22].

Hydrogels, by creating a supportive environment for cell growth, provide necessary nutrients and factors through the culture medium. Although various strategies exist for manufacturing hydrogels, the formation of microspheres is a widely used method, allowing optimal isolation of individual cells and subsequent applications [22]. It is crucial to note that the application of hydrogels can vary based on their composition, including different cell lines or the specific factors and drugs to which cells will be exposed.

The combination of techniques, such as the manufacture of biocompatible compounds and microfluidic technology, results in the production of micro-hydrogels with versatile applications not only in cellular or molecular biology but also in drug delivery systems, both passive and active, and the mixing of chemical components and nanocomposites [23,24,25].

Similarly, the integration of low-cost technology for hydrogel manufacturing with the production of droplets enables the isolation of gene-edited cells and the generation of stable lines. This work leverages the efficiency of CRISPR-Cas9 technology in conjunction with droplet manufacturing to achieve more efficient clonal selection than conventional methods involving antibiotic selection and cell concentration dilution. The focus of this study is on modifying hiPSCs, selecting them using extracellular matrix hydrogels, enhancing the efficiency of clonal selection for the stable line modified by CRISPR, and significantly reducing the time required for establishing the line through long-term single-cell culture.

## 2. Materials and Methods

### 2.1. Design and Fabrication

Microfluidic device was designed for (Layout design editor software). The PDMS microdevices (Sylgard 184, Dow Corning Marietta, Georgia, USA,) were manufactured as described previously [26]; in the case of the forming device of droplets. Briefly, a mold in high relief with the desired design was made by photolithography in a 700 µm thick silicon wafer (Virginia Semiconductor, Inc., Fredericksburg, VA, USA) using the negative resin SU-8 (MicroChem, Newton, MA, USA). The microchannels have a final height of 150 µm. Next, the mold was placed under vacuum with trichloro (1H, 1H, 2H, 2H-perfluoro-octyl) silane (Sigma, St. Louis, MO, USA) for 1 h to protect the SU-8 resin from detachment by releasing PDMS from the mold. The PDMS was mixed with the curing agent in a 10:1 ratio, and the mixture was placed under vacuum for 1 h to remove air bubbles. Then, the mixture was poured back under vacuum for 1 h and cured in an oven at 70 °C for 70 min. The PDMS was not molded, and the fluidic connection ports were constructed by drilling holes in the PDMS with a syringe needle (21-gauge, internal diameter of 0.51 mm). Finally, the PDMS was assembled with the glass base. Through the plasma oxygen system (deposition of chemical vapors enhanced with plasma), the device and the glass base are exposed for 3 min at a pressure of more than 4000 g overnight [18,19,20]. In the case of a droplet storage chip, multilevel photopolymer technology and microdevice manufacturing technology were used, as described in previous work [25,26,27].

**Figure 1:**
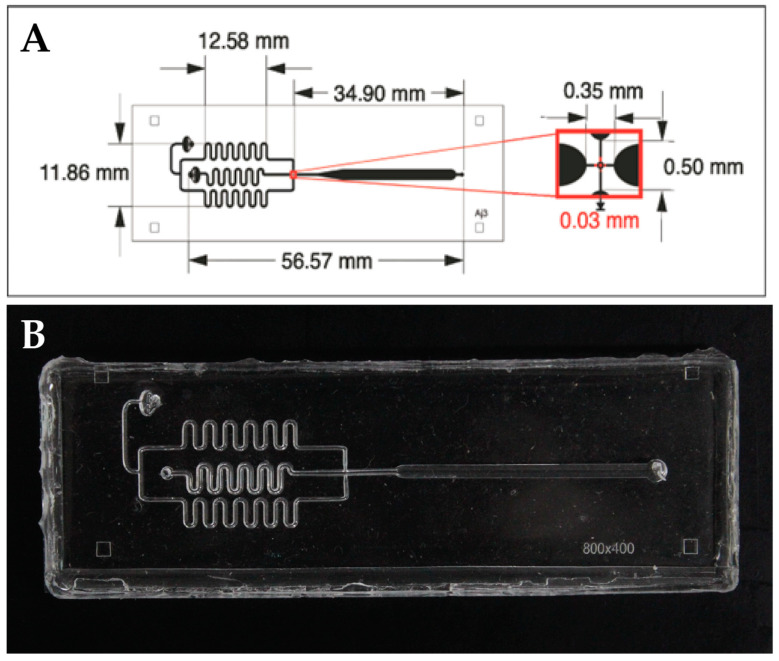
(**A**) Scheme of the droplet-forming microdevice. (**B**) Image of real microdevice.

Figure 1 shows in detail the measurements of the droplet-forming microdevice.

### 2.2. Modified HiPSC Line

The hiPSC used was a modified line expressing the fluorescent protein mCerulean–H2B, introduced via plasmid (Addgene plasmid #55375; http://n2t.net/addgene:55375 accessed on 1 November 2023; RRID:Addgene_55375, with its sequence detailed in Supplementary Information S2). This allowed us to observe the behavior and subsequent knockout due to the loss of fluorescence. Cells were routinely cultured in E8-Flex on Geltrex with ROCK inhibitor, all from Thermo Fischer Scientific. All cultures were maintained at 37 °C in an atmosphere saturated with 95% air and 5% CO_2_. Cell concentrations for the cell line maintenance were up to 1,200,000 cells/mL in 6-well multiwell plates, and for transfection and corresponding editing in 12-well multiwell plates, concentrations were at 200,000 cells/mL.

### 2.3. Design of the Guides and Flanking Primers

**Figure 2:**
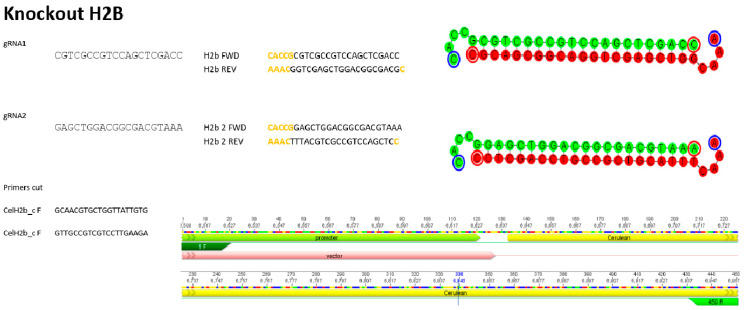
Guides used to knock out the H2B–Cerulean and primers for verification.

For this study, a couple of guides were designed with the help of the online benchling/CRISPR tool, where the ones with the highest percentage of in silico effectiveness were chosen to knock out the mCerulean–H2B protein. In addition, primers flanking the region of interest were designed to subsequently verify their effectiveness in inhibiting the fluorescent protein (Figure 2).

### 2.4. Production of Droplets and Hydrogels

The production of droplets and monodisperse hydrogels involved the use of the biocompatible oil FluoroSurfactant-HFE7500 at a concentration of 5% wtH. In both cases, the mixture of the plasmid with the guides, along with a combination of mTser and Optimen media, and Lipofectamine (Thermo Fisher Scientific, Carlsbad, CA, USA^®^) were used as the dispersed phase.

For the hydrogel manufacturing process, a variant of commercial extracellular matrix (geltex^®^) was utilized at varying concentrations ranging from 40X to 100X to improve polymerization and minimize channel clogging issues. Additionally, the temperature for assembling the hydrogel production system was maintained at 8 °C.

The flow rates and manufacturing protocol for the droplets were adapted from reference [28]. For cell isolation using extracellular matrix hydrogels, the flow rates of the continuous phase were adjusted to 6.5 μL/min, while the dispersed phase was set to 5 μL/min, ensuring stable production.

### 2.5. Image Analysis

The analysis and quantification of results obtained in the experimental phases were conducted using the IMAGE J program and specific macros (TrackMate version v3.5.1, with specific modifications). This was performed for the quantification of transfection efficiency in single-cell analysis. Images were captured using the EVOS^®^ 2 FL adapted fluorescence microscope, totaling 126 images taken directly in the multiwell seeding plates. A total of 430 cells were analyzed, encompassing both control wells and hydrogels formed by the microfluidic system.

## 3. Results

### 3.1. Editing Efficiency by the CRISPR-Cas9 Method in Droplets

As a first step, the efficiency of single-cell editing was compared using the CRISPR-Cas9 method with the guides initially designed to target the fluorescent protein H2B–mCerulean, which was inserted into the hiPSCs and transformed into the stable line. Individual cells were encapsulated in microdroplets containing medium and the guides we designed in advance within the base plasmids. A plasmid concentration of 0.7 μg/μL was used at the time of transfection, and this concentration was employed in both the conventional method and the microdroplet-forming microdevice injection.

To enhance cell survival and achieve the optimal timeframe for guide action on the protein, we relied on standardized times in single-cell gene editing methods, as cited by Perez-Sosa et al., 2022 [29]. The optimal time in droplets was set at 8 h, reaching a maximum of 24 h. It is noteworthy that the control in the plate was exposed to the plasmid and transfection agents for 24 h. An initial cell concentration of 200,000 cells/mL was used.

**Figure 3:**
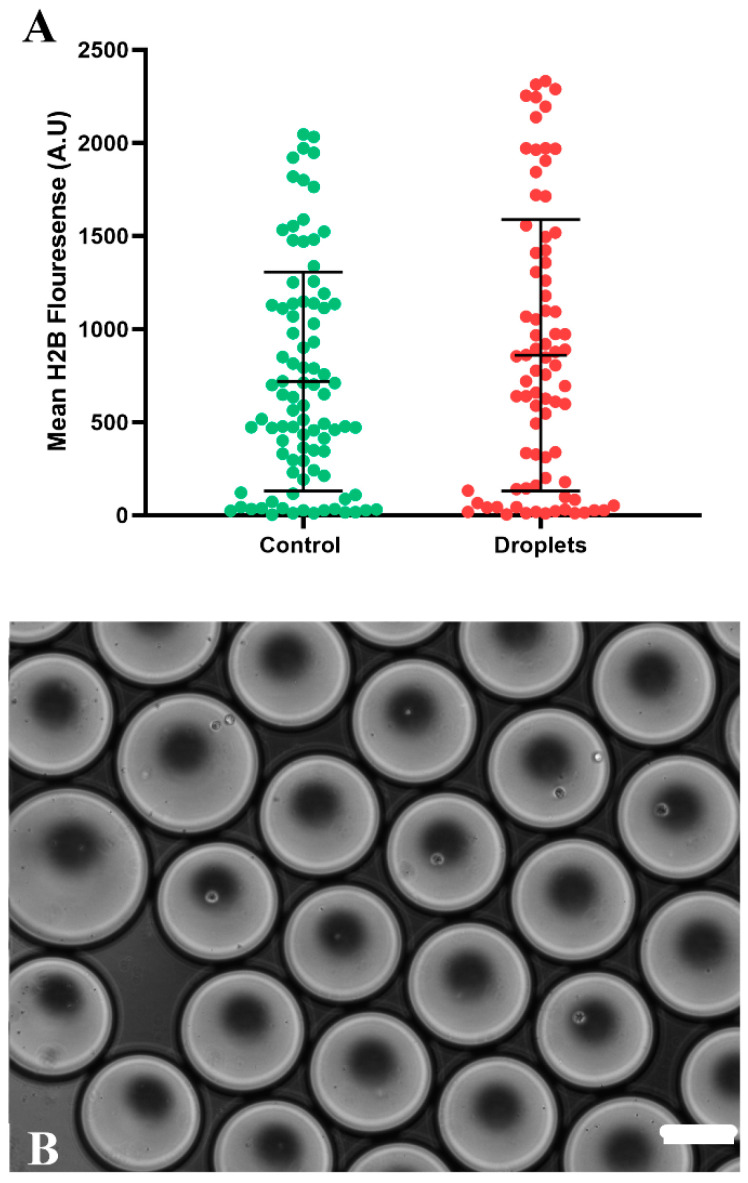
(**A**) Shows the comparison of the knockout of the H2B–mCerulean protein by the CRISPR-Cas9 method in droplets (red) and culture dish (green). A total of 246 individual cells were analyzed, including both the control group and the droplets, with a standard deviation of 4.16644. (**B**) Representative images of cells that were edited by the CRISPR-Cas9 method within droplets. Scale bar corresponds to 200 μm.

To measure the efficiency of our method, we assessed the fluorescence that cells were losing using the ImageJ tool and specific plugins. As shown in Figure 3, it was possible to eliminate the protein using the designed guides; in our case, we designed a pair of guides (sgRNAs) (Figure 2). The reduction in fluorescence began to be observed 4 h after transfection, reaching the lowest point 24 h after the process began, both in encapsulated cells and in the controls of the conventional method.

Both guides showed similar efficiency, although they observed better efficiency in gRNA 2 for breaking down the protein and eliminating the fluorescence produced by H2B–mCerulean. However, compared to the control plate, the efficiency was very similar in both the microdroplets and the plate. This demonstrated that the CRISPR-Cas9 single-cell editing system is effective in both controls and produced microdroplets.

### 3.2. Clonal Selection Using Extracellular Matrix Hydrogels

**Figure 4:**
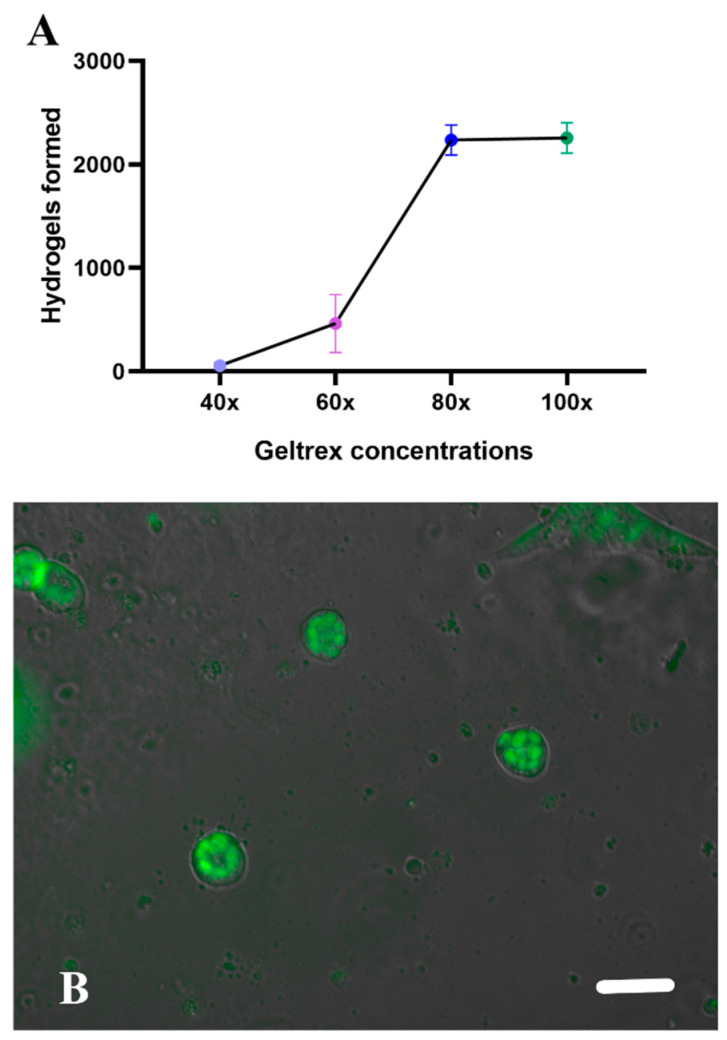
Hydrogel formation. (**A**) Shows the comparison of geltrex concentrations in the formation of hydrogels. 40X in light blue, 60X in purple, 80X in blue, 100X in green. (**B**) Representative image of the hydrogels in a conventional culture plate. Scale bar corresponds to 200 µm.

We proceeded to isolate the cells on hydrogels, basing our procedure almost entirely on the established protocol for droplet formation with fluorinated oil and culture medium [30]. However, in this case, the culture medium was substituted with the extracellular matrix component. Various dilutions were experimented with to determine the most effective concentration for polymerization and the formation of monodisperse hydrogels (see Figure 4A). The 80x concentration proved optimal for both formation and polymerization, facilitating the injection of flows into the microdevice. It is important to note that, to generate droplets from this type of hydrogel, the entire system, including hoses, the device, pipette tips, and even the entire culture room, needed to be cooled to approximately 8 °C. This precaution was taken to prevent the geltrex^®^ from polymerizing in the hoses and inside the droplet-forming device.

Following the formation of hydrogels, they were collected in a 1.5 mL volume microcentrifuge tube and incubated for 45 min under conventional culture conditions at 37 °C with an atmosphere of 95% humidity and 5% CO_2_. These conditions were maintained to minimize stress on the cells and ensure the best possible viability over time. This allowed the cell viability to remain stable both in the pre-transfection culture (96%) and after transfection and knock-out of the H2B–Cerulean protein (94%).

After polymerization, the hydrogels were released from the fluorinated oil and surfactant using the PFO protocol. The purpose of this step was to grant the cells access to the culture medium and growth factors over time.

**Figure 5:**
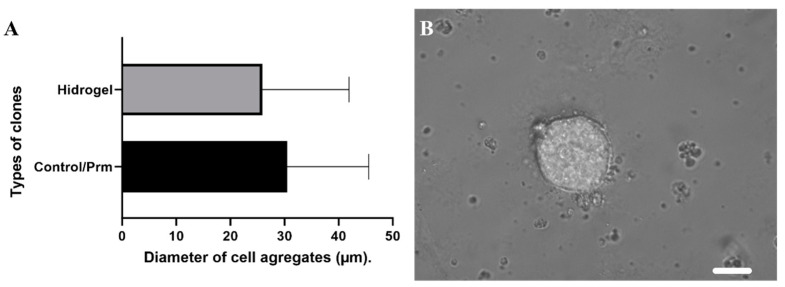
Cell aggregates. (**A**) Shows the comparison of the growth of the hydrogels vs. the colonies selected by the conventional method. (**B**) Representative image of hydrogel cell aggregates at 72 h after seeding. Scale bar is 200 µm.

At the end of the process, the clonal selection took place between 72 and 96 h of hydrogel growth. This is to guarantee the robust selection of clones that lost fluorescence due to the action of knocking out the H2B–mCerulean that they previously possessed. Hydrogels were measured in comparison to the conventional dilution method in Figure 5. Although the control cell aggregates had larger diameters, clonal selection was facilitated using hydrogels by isolating individual clone cells from specific points within the hydrogel. This approach allowed us to avoid selection based on antibiotics and resulted in increased efficiency and yield of the clones in terms of time.

This selection was given in a P60 culture dish to facilitate the isolation of the hydrogels onto new culture plates.

### 3.3. Molecular Verification of the Knockout of H2B–mCerulean

**Figure 6:**
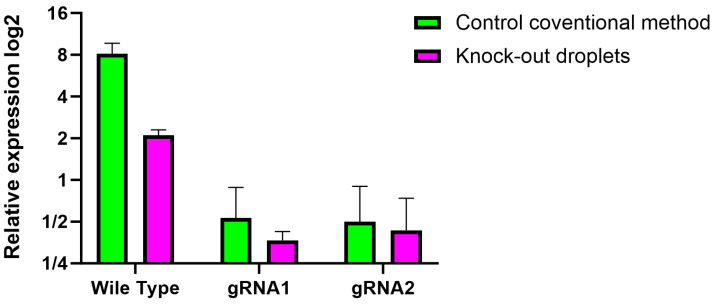
The results of the qPCR are shown, analyzing the two guides used and the wild-type phenotype without modification by CRISPR-Cas9.

As a final check that our lines were clonal and knocked out H2B–mCerulean, a round of qPCR was performed on several of the randomly selected hydrogels. Even though fluorescence was no longer seen, this indicated that the knockout of the protein in question was successful; these tests were carried out, as shown in Figure 6.

This verification convincingly demonstrated the efficiency of our CRISPR-Cas9 droplet system in carrying out specific modifications in isolated individual cells. Furthermore, it was verified that generating stable lines through hydrogels can be a robust alternative for reducing the time needed for clonal selections with antibiotics and the growth of colonies and cell aggregates. This is particularly effective when a reporter is available to accurately select and isolate the edited cells.

## 4. Conclusions

This present work successfully demonstrated the feasibility of genetically editing single cells by isolating them through droplets and selecting them using extracellular matrix hydrogels in induced pluripotent stem cells (hiPSCs) through the utilization of the CRISPR-Cas9 technique. Our innovative method not only achieves successful genetic editing but also stands out for its cost-effectiveness and versatility when compared to other similar methods [31]. Its adaptability to different cell lines and compatibility with both automatic and semi-automatic work systems make it a promising approach for diverse applications.

The utilization of droplets and hydrogels, produced through the same technique, showcased high yields in isolating single cells. Furthermore, the application of hydrogels has proven to be beneficial for the long-term culture of single cells, ensuring their viability over time. This extended viability facilitates the selection and segregation of cells, ultimately leading to the establishment of a stable cell line post-knockout of the H2B–mCerulean protein.

The integration of techniques such as droplet production and gene editing represent a potent tool with vast potential across various domains, including personalized medicine, targeted drug delivery, and cell-based therapies. Despite these promising outcomes, it is important to acknowledge that challenges persist in establishing clonal selection in single cells using microfluidic tools as a routine laboratory procedure. This work contributes to this field by providing evidence that greater efficiency and time reduction can be achieved in the future through continuous optimization and refinement of this innovative procedure.

In conclusion, our findings not only showcase the success of our methodology in achieving precise genetic edits in single cells but also highlight the broader implications and future possibilities of this technique in advancing applications across diverse biomedical fields. As we continue to address challenges and refine our approach, we anticipate that our method will play a pivotal role in shaping the future of single-cell genetic editing technologies.

## Data Availability

Data are contained within the article and Supplementary Materials.

## Supplementary Materials

The following supporting information can be downloaded at: https://www.mdpi.com/article/10.3390/mi15030413/s1, FIJI code for Image Analysis and Plasmid sequence H2B-Cerulean.
