# Supplementary material for: Droplets for Gene Editing Using CRISPR-Cas9 and Clonal Selection Improvement Using Hydrogels
Source: Micromachines (Basel). 2024 Mar 19;15(3):413. doi: 10.3390/mi15030413 (PMC10972466; doi:10.3390/mi15030413)
Supplement: Supplementary file 1 [file micromachines-15-00413-s001.zip › micromachines-2769677-supplementary.pdf]

micromachines

MDPI

Supplementary Information

Droplets for gene editing using CRISPR-Cas9 and clonal selection improvement using hydrogels.

Camilo Pérez-Sosa [1], Maximiliano. S. Pérez [1, 2, 5], Alexander Paolo Vallejo-Janeta[1], Shekhar Bhansali[4], Santiago Miriuka [3] and Betiana Lerner [1, 4,5]

1 IREN Center, National Technological University, Buenos Aires 1706, Argentina[2]
2 Institute of Biomedical Engineering, Buenos Aires University (UBA), Buenos Aires, C1063ACV, Argentina
3 National Council for Scientific and Technical Research - Foundation for the Fight against Neurological Diseases of Childhood, (LIAN-CONICET-FLENI), FLENI Sede Escobar, Ruta 9 Km 53, 1625, Belén de Escobar, Buenos Aires, Argentina
4 Department of Electrical and Computer Engineering, Florida International University, Miami, Florida 33174, US
5 Collaborative Research Institute Intelligent Oncology (CRIION), Hermann-Herder-Straße 4, 79104 Freiburg im Breisgau, Germany
* Correspondence: belerner@fiu.edu; maxperez@fiu.edu

Code FIJI

channel_segment = 1; median_radius = 4; threshold_method = "Otsu"; min_particle_size = 10;

// Get the title of the image original_image = getTitle(); // Run all the preprocessing needed. Like setting the measurements, closing images, cleaning ROIs runPreprocessing();
// This does the actual segmentation of the data runSegmentation(channel_segment, median_radius, threshold_method);
96 //
This produces the results analyzeParticles (original_image, min_particle_size); // The postProcessing script creates the output image so that we may check the results postProcessing(original_image);

// Preprocessing function to prepare Fiji's environement to analyze the image function runPreprocessing() { // Some preprocessing // Make sure there are no ROIs in the ROI manager roiManager("Reset");

// Ensure we are computing the right results, making sure that // the label is displayed and that we keep the stack position information run("Set Measurements...", "area mean shape stack display redirect=None decimal=3"); // We explicitely do not close the results table because

// we want the results to be appended for each image that will be analysed // hence the warning above // Close all other images, to reduce clutter close("\ \Others"); }

// This filters and segments the image so that we get a binary mask at the end function runSegmentation(channel_segment, median_radius, threshold_method) { 97 // Run the segmentation.

// Duplicate the first channel (DAPI)

// Filter the image with a median filter and reduce intensity variation by taking the square root // Set an auto-threshold and get a mask // Clean the mask with a median filter and a watershet run("Duplicate...", "title=DAPI duplicate channels="+channel_segment); run("Median...", "radius="+median_radius);
run("32-bit"); run("Square Root"); run("Enhance Contrast", "saturated=0.35"); setAutoThreshold(threshold_method+" dark no-reset");
//setAutoThreshold("MaxEntropy dark no-reset"); setOption("BlackBackground", true); run("Convert to Mask"); run("Median...", "radius=2"); //run("Watershed"); // End of segmentation

Figure S1. Detail of the sequence used in IMAGEJ for single cell analysis and clone growth.

Supplementary information 2

Sequence plasmid H2B- Celurian

ACGTCAGATCCGCTAGCGCTACCGGTCGCCACCATGGTGAGCAAGGGCGAGGAGCTGTTCAC-
CGGGGTGGTGCCCATCCTGGTCGAGCTGGACGGCGACGTAAACGGCCACAAGTTCAGCGTGTCCGGCGAGG
GCGAGGGCGATGCCACCTACGGCAAGCTGACCCTGAAGTTCATCTGCACCACCGGCAA-
GCTGCCCGTGCCCTGGCCCACCCTCGTGACCACCCTGACCTGGGGCGTGCAGTGCTTCGCCCGCTACCCCGAC
CACATGAAGCAGCACGACTTCTTCAAGTCCGCCATGCCCGAAGGCTACGTCCAGGAGCGCAC-
CATCTTCTTCAAGGACGACGGCAACTACAAGACCCGCGCCGAGGTGAAGTTCGAGGGCGACACCCTGGTGA
ACCGCATCGAGCTGAAGGGCATCGACTTCAAGGAGGACGGCAACATCCTGGGGCACAAGCTG-
GAGTACAACGCCATCAGCGACAACGTCTATATCACCGCCGACAAGCAGAAGAACGGCATCAAGGCCAACTT
CAAGATCCGCCACAACATCGAGGACGGCAGCGTGCAGCTCGCCGACCACTACCAG-
CAGAACACCCCCATCGGCGACGGCCCCGTGCTGCTGCCCGACAACCACTACCTGAGCACCCAGTCCAAGCTG
AGCAAAGACCCCAACGAGAAGCGCGATCACATGGTCCTGCTGGAGTTCGTGACCGCCGCCGG-
GATCACTCTCGGCATGGACGAGCTGTACAAGTCCGGACTCAGATCTGGAGGCTCCGGAGGCCCAGAGCCAG
CGAAGTCTGCTCCCGCCCCGAAAAAGGGCTCCAAGAAGGCGGTGACTAAGGCGCAGAA-
GAAAGGCGGCAAGAAGCGCAAGCGCAGCCGCAAGGAGAGCTATTCCATCTATGTGTACAAGGTTCTGAAGC
AGGTCCACCCTGACACCGGCATTTCGTCCAAGGCCATGGGCATCATGAATTCGTTTGTGAAC-
GACATTTTCGAGCGCATCGCAGGTGAGGCTTCCCGCCTGGCGCATTACAACAAGCGCTCGACCATCACCTCC
AGGGAGATCCAGACGGCCGTGCGCCTGCTGCTGCCTGGGGAGTTGGCCAAGCAC-
GCCGTGTCCGAGGGTACTAAGGCCATCACCAAGTACACCAGCGCTAAGTAAGGATCCACCGGATCTAGATA
ACTGATCATAATCAGCCATACCACATT.
